# DeloRes trial: study protocol for a randomized trial comparing two standardized surgical approaches in rectal prolapse - Delorme’s procedure versus resection rectopexy

**DOI:** 10.1186/1745-6215-13-155

**Published:** 2012-08-29

**Authors:** Simone Rothenhoefer, Florian Herrle, Alexander Herold, Andreas Joos, Dieter Bussen, Meinhard Kieser, Petra Schiller, Christina Klose, Christoph M Seiler, Peter Kienle, Stefan Post

**Affiliations:** 1 Department of Surgery|, University Medical Centre Mannheim (UMM), University of Heidelberg, Heidelberg, Germany; 2 Institute of Proctology Mannheim (EDZ), Mannheim, Germany; 3 Institute of Medical Biometry and Informatics, University of Heidelberg, Heidelberg, Germany; 4 Study Centre of the German Surgical Society, University of Heidelberg, Heidelberg, Germany

**Keywords:** Rectal prolapse, Surgery, Delorme’s procedure, Resection rectopexy, Recurrence

## Abstract

**Background:**

More than 100 surgical approaches to treat rectal prolapse have been described. These can be done through the perineum or transabdominally. Delorme’s procedure is the most frequently used perineal, resection rectopexy the most commonly used abdominal procedure. Recurrences seem more common after perineal compared to abdominal techniques, but the latter may carry a higher risk of peri- and postoperative morbidity and mortality.

**Methods/Design:**

DeloRes is a randomized, controlled, observer-blinded multicenter trial with two parallel groups. Patients with a full-thickness rectal prolapse (third degree prolapse), considered eligible for both operative methods are included. The primary outcome is time to recurrence of full-thickness rectal prolapse during the 24 months following primary surgery. Secondary endpoints are time to and incidence of recurrence of full-thickness rectal prolapse during the 5-year follow-up, duration of surgery, morbidity, hospital stay, quality of life, constipation, and fecal incontinence. A meta-analysis was done on the basis of the available data on recurrence rates from 17 publications comprising 1,140 patients. Based on the results of a meta-analysis it is assumed that the recurrence rate after 2 years is 20% for Delorme’s procedure and 5% for resection rectopexy. Considering a rate of lost to follow-up without recurrence of 30% a total of 130 patients (2 x 65 patients) was calculated as an adequate sample size to assure a power of 80% for the confirmatory analysis.

**Discussion:**

The DeloRes Trial will clarify which procedure results in a smaller recurrence rate but also give information on how morbidity and functional results compare.

**Trial registration:**

German Clinical Trial Number DRKS00000482

## Background

Full-thickness prolapse of the rectum is defined as protrusion of the full thickness of the rectal wall through the anus. There is a peak in the incidence of this disease in the seventh decade of life. A total of 80% to 95% of the patients are women [1,2]. The etiology of rectal prolapse is still unknown. Fifty percent to seventy percent of patients complain of longstanding constipation, the rate of fecal incontinence ranges between 40% and 80% [3-6]. Other predisposing conditions include chronic straining defecation, pregnancy, previous surgery, and neurological disease. There is probably a genetic disposition to this condition.

Treatment of rectal prolapse is usually surgical. More than 100 surgical approaches have been described in the literature. The aims of the operation are to correct the rectal prolapse, to restore normal bowel function, and to avoid a recurrence of full-thickness rectal prolapse. For many patients, constipation and fecal incontinence improve after surgery [4,7,8].

Basically, two different surgical approaches can be distinguished: perineal and transabdominal procedures. In general, perineal surgical repairs supposedly cause less morbidity and mortality compared with abdominal operations [7,9,10]. They are considered especially indicated in the elderly and/or high-risk patients as they can also be done in spinal or epidural anesthesia [9,11,12]. On the other hand, recurrences seem more frequent after perineal techniques than after abdominal operations [1-3,13]. Abdominal repairs involve fixing the rectum to the sacrum by using either mesh or sutures. In addition to rectopexy a sigmoid resection is commonly performed. Laparoscopic repair of rectal prolapse has similar morbidity and recurrence rates as open surgery [7,14]. There are data suggesting that the laparoscopic compared to the conventional approach has short-term benefits as described for other laparoscopic abdominal procedures (reduced length of hospital stay, less postoperative pain, faster return to normal bowel function, and less wound complications) [9,15]. But when using a modern fast-track regime (involving multimodal balanced anesthesia and analgesia including peridural catheter, early normal nutrition and mobilization as well as avoidance of tubes and catheters) the advantages of a laparoscopic approach have become somewhat less clear [15-17]. In general the fast-track regime allows enhanced recovery and discharge of patients compared to conventional postoperative management. Application of such a fast-track regime allows compensation of assumed disadvantages like more pain or slower mobilization of patients after open abdominal surgery compared to laparoscopic surgery.

In conclusion, perineal as well as abdominal (mostly laparoscopic) procedures remain the established options in the surgical treatment of rectal prolapse without evidence from adequately designed randomized studies which of these procedures are superior. There are only insufficient data available on the long-term benefits and risks, quality of life, and total treatment costs. Our trial aims to contribute to the very scarce high-level-evidence basis of randomized trials comparing a perineal *vs*. an abdominal approach. The urgent need for a randomized trial such as DeloRes was also pointed out in the Cochrane Review by Tou in 2009 [18].

## Methods/Design

### Trial design

DeloRes is a randomized, controlled, observer-blinded multicenter trial with two parallel groups.

### Patient population

#### Inclusion criteria

• Full-thickness rectal prolapse, externally visible on straining

• ASA-Score I-III

• Patient is suited for both standardized surgical approaches

• Patient is able to cooperate

 · Patient has given written informed consent

• Aged 18 years or older

#### Exclusion criteria

• Recurrence of full-thickness rectal prolapse

• Patient with stoma

• Patient with inflammatory bowel disease

• Pregnancy or breastfeeding

• Patient currently under chemotherapy

• Active malignant disease and life expectancy less than 24 months

• Body mass index greater than 40

• Participation in another intervention trial with interference of intervention and outcome of this study

The aim was to exclude as few patients as possible, but nevertheless minimize attrition bias in long-term follow-up and enable a high recruitment rate given the fact that patients presenting with full rectal prolapse are a small population of a reasonably higher population of patients not willing to present at their physicians because of misinformation or shame.

Thus patients with recurrent rectal prolapse would often not be acceptable for surgeons to be randomized to perineal vs. abdominal approach especially if the prior surgery had been a perineal approach. Patients not surviving at least 24 months would not be available for the primary endpoint visit at 24 months postoperatively. And patients with BMI > 40 had been excluded as they are very few and do not represent the common patient habitus of rectal prolapse.

### Scheme of intervention

DeloRes will be performed in 13 trial centers. All participating centers are cooperating units with a special focus on colorectal surgery and all centers perform between eight and sixty surgical interventions for rectal prolapse per year. In order to minimize performance bias, only surgeons with an adequate experience in performing the approaches investigated operate on patients in this study (minimum number of procedures performed (‘life-time experience’): Delorme’s procedure=20, (laparoscopic) resection rectopexy=20). Each study center provides at least one surgeon with the required experience for Delorme’s procedure and one surgeon with the required experience for (laparoscopic) resection rectopexy (expertise based, best team approach). To ensure standardization for all participating centers an operative manual with standardized video sequences will be prepared. Moreover, consented crucial steps of each procedure will be captured in the clinical record file. In addition, in order to allow confirmation of a certain procedure quality, selected digital photos will be taken.

To reflect current practice and to increase external validity of the trial, perioperative standards (for example, bowel preparation, parenteral nutrition, fast-track, postoperative application of laxatives) will not be strictly standardized. Each trial center, however, will document its own standards in a short questionnaire and agrees not to change this standard during trial recruitment.

### Recruitment and trial timeline

Recruitment of participants started in September 2010. The duration of the trial for each patient is expected to be 60 months with follow-up at 6, 12, 24, and 60 months after the primary surgery.

### Treatment assignment and randomization

Consecutively screened and eligible patients will be included in the trial. In order to achieve comparable intervention groups, patients will be allocated by preoperative randomization 24 to 48 h prior to surgery after given written informed consent using a centralized web based tool ( http://www.randomizer.at). Block randomization stratified by center will be applied to achieve equal group sizes per center.

### Interventions and trial flow

**Figure 1:**
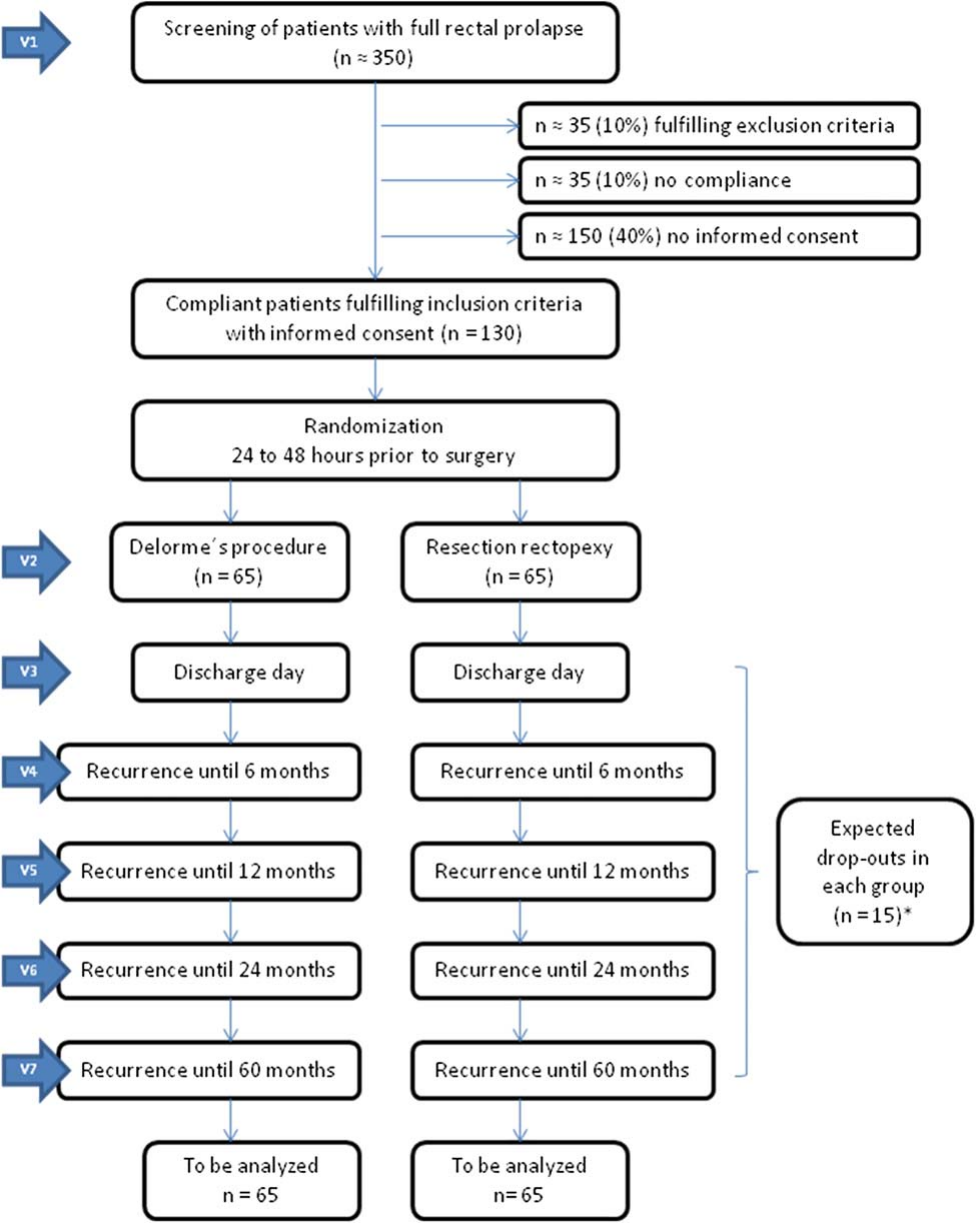
Trial flow/intervention scheme.

The laparoscopic approach is considered the standard approach for the transabdominal operation of full-thickness rectal prolapse. Laparoscopic resection rectopexy is suggested to yield better results in the early postoperative phase in comparison to the open approaches with equal recurrence rates in comparison to the open approach. However, the advantages of a laparoscopic approach become somewhat less evident under a fast-track regime, therefore open resection rectopexy is also acceptable via a Pfannenstiel incision. The latter approach has proven to be only moderately invasive in numerous procedures of the lower abdomen. The most frequently used perineal procedure is Delorme’s procedure, which shows low postoperative morbidity. The trial flow is illustrated in Figure 1.

#### Description of trial days

All patients will be informed about the purpose of the trial and surgical treatment modalities comprising their benefits as well as risks. The schedule of trial interventions is presented in Table 1.

**Table 1:** Study visits of DeloRes

	**Visit 1**	**Visit 2**	**Visit 3**	**Visit 4**	**Visit 5**	**Visit 6**	**Visit 7**
	**Screening**	**Surgery**	**Day of Discharge**	**6 months after Surgery**	**12 months after Surgery**	**24 months after Surgery**	**60 months after Surgery**
Demographic and clinical baseline data	×						
Inclusion/Exclusion	×						
Randomization	×						
Intervention		×					
Assessment of Complications and Safety		×	×	×	×	×	×
Recurrence				×^a^	×^a^	×	×
Secondary endpoints		×	×	×^a^	×^a^	×	
SF-12	×			×^a^	×^a^	×	
Herold constipation score	×			×^a^	×^a^	×	
Wexner score	×			×^a^	×^a^	×	
Rockwood-FIQLScore	×			×^a^	×^a^	×	

^a^It is intended that patients come to the trial sites for all follow-up visits. If they are unable to travel they will be visited by a study nurse. At the 6- and 12-month follow-up visits, recurrence status, secondary endpoints can exceptionally be assessed by phone interview and/or by mailing questionnaires.

### Outcome measures and definitions

The clinically most relevant parameter in the treatment of full-thickness rectal prolapse is the time to recurrence. Therefore this parameter is defined as primary outcome measure, evaluated during the 24 months following primary surgery. Recurrence is defined as the circular protrusion of rectal mucosa through the anal canal and will be examined by standardized history, clinical examination, and photo documentation. To assess the primary outcome, patients are asked to perform a straining maneuver in a sitting position (patients not able to sit will be assessed in a lateral decubitus position). Then a photograph is taken during straining. The primary outcome is finally determined by the independent review board on the basis of the photograph.

Secondary endpoints are time to and incidence of recurrence of full-thickness rectal prolapse during 5-year follow-up, duration of surgical intervention, morbidity, SAE, all causes of mortality within 3 months after primary surgery (this period was chosen to get any mortality possible related to surgery. Patients with rectal prolapse are usually rather old and frail. There are multiple reasons for further morbidity or mortality which are expected to be unrelated to the surgical procedure in case they occur beyond the first 3 months), length of initial hospital stay, reoperation rate for recurrent prolapse, cumulative hospital stay and change in quality of life, constipation, and fecal incontinence as measured by appropriate questionnaires.

The time to and the incidence of recurrence of full-thickness rectal prolapse during a 5-year follow-up after primary surgery will be assessed as explained above. Morbidity occurring during the primary hospital stay is further differentiated into surgical and non-surgical complications, which are specifically defined as following: severity grading is done with the Clavien classification [19] and for anastomotic leakage with the classification of the International Study Group of Rectal Cancer [20]. The definitions of the secondary outcomes are shown in Table 2. 

**Table 2:** Definitions of secondary outcomes

***Surgical complications***	
Postoperative ileus	Obstructive or paralytic symptoms after surgery with the need to suspend food intake and/or insert a gastric tube; this has to be confirmed radiologically (by abdominal sonography or plain abdominal X-ray or CT scan)
Postoperative hemorrhage	Need for administration of two or more red cell concentrates within the first 24 h postoperatively or need for reoperation
Surgical site infection	CDC definition [21]
Intra-abdominal abscess	Intra-abdominal collection of purulent or infected fluid (confirmed by culture) confirmed by puncture or by surgical reintervention
Anastomotic leakage	Grade A-C, Definition of grade according to International Study Group of Rectal Cancer 2010, diagnosed by CT scan with radiographic enema, endoscopy, drain secretion (stool) or reoperation
***Non-surgical complications***	
Thrombosis	Clinical evidence (for example, pain, swelling, warmth, erythema) of a leg or pelvic vein thrombosis confirmed by duplex sonography or CT angiography, which was not previously known
Pulmonary embolism	Clinical (for example, tachycardia, dyspnea) and/or radiological evidence of pulmonary embolism confirmed by spiral CT or lung perfusion scintigram
Postoperative pulmonary infection	At least three of the following:
	- temperature > 38°C
	- purulent tracheal secretion
	- leucocytes >12 or < 4.5 [10E9/L]
	- elevated CRP
	AND Evidence for pulmonary infection radiologically
Renal failure	Need for dialysis or hemofiltration
Cerebral insult	Ischemic or non-ischemic cerebrovascular event with persistent paresis or paralysis without previous history confirmed by CT or MRT
Myocardial infarction	Electrocardiogram (NSTEMI or STEMI) and enzyme (Troponin I) changes suggestive of myocardial infarction or needing admission to coronary care unit
*Serious adverse events*	SAE occurring from the day of randomization until regular end of trial follow-up or withdrawal
*Mortality*	All causes of mortality within 3 months after primary surgery
*Duration of surgical intervention*	First incision to the completion of skin closure (resection rectopexy) or the last coloanal suture (Delorme’s procedure)
*Length of initial hospital stay*	Means the hospitalization period from the primary operation date until the day of discharge (= postoperative hospital stay)
*Reoperation rate for recurrent prolapse*	Any surgical intervention for a recurrence
*Cumulative hospital stay*	Defined by the days in hospital after the primary surgery as well as the following hospitalizations due to complications or recurrence within 24 months after the primary operation
*Change of quality of life, constipation and incontinence*	
	Measured by means of the appropriate questionnaires SF-12, ( [22], validated in German), Herold constipation score ( [23], evaluated in German, but not yet published), Wexner-Score ( [10], non-validated in German, most commonly used score) and Rockwood-FIQLScore ( [24], validated), prior to the surgical intervention as well as 6, 12, and 24 months after the intervention

### Data management

All protocol-required information collected during the trial must be documented in the paper-based CRF by the investigator. Any outstanding entries must be completed immediately after the final examination. The completed CRF must be reviewed and signed by the investigator named in the trial protocol.

In order to ensure that the database reproduces the CRFs correctly a double entry of data will be performed by two different people.

### Compliance/rate of loss to follow-up

To reduce the number of patients not able or willing to visit the study centers a study nurse will be appointed to visit the patients and to assess the outcome at the specified time (‘flying study-nurse’). Furthermore, the time-to-event method will be applied for the primary analysis to allow for all available information including the observation time of patients who drop out early. Nevertheless, an additional percentage of patients will be recruited to assure a sufficient power for the confirmatory analysis.

### Safety assessments and reporting of adverse events

Analysis of safety-related data is performed with respect to frequency of serious adverse events, frequency of serious adverse events stratified by intensity and causality, and frequency of morbidity in both treatment groups.

From the day of randomization until the regular end of trial follow-up or until premature withdrawal of the patient, all serious adverse events must be documented on a ‘serious adverse event form’ available in the investigator site file.

A serious adverse event will be defined as an event that results in death, is immediately life-threatening, requires or prolongs hospitalization beyond the 20th postoperative day for any medical reason, requires readmission for any medical reason within 3 months after the operation within this trial, requires readmission for treatment of surgical complications related to the study operation beyond 3 months postoperatively, or results in persistent or significant disability or incapacity. Serious adverse events will be classified in intensity (mild, moderate, severe), outcome (recovered completely, recovered with sequelae, not recovered, death, unknown), and causality (unrelated, possibly related, probably related, definitely related, not assessable).

The assessment is based on surgical findings and the clinical course of the patient. It needs to be done by the investigators. Serious adverse events have to be reported by the attending physician to the coordinating investigator or one of both study supervisors within 24 h after the SAE becomes known.

### Statistical methods

#### Sample size calculation

The sample size calculation is based on the analysis of the primary endpoint (time to recurrence of full-thickness rectal prolapse during a 2-year follow-up). A meta-analysis including 17 publications (Delorme [2,5,6,8,9,12,13,25]; (Laparoscopic) Resection Rectopexy [1,3,4,7,14,26-28]) showed recurrence rates ranging from 10% to 30% (Delorme’s procedure) and from 0% to 11% (laparoscopic resection rectopexy) (Table 3). Based on these data, we assume a recurrence rate 2 years after randomization of 20% for the Delorme’s procedure and 5% for resection rectopexy, respectively. If the unstratified two-sided log rank test at level of significance α=5% was applied 2 x 50=100 patients would be required to achieve a power of 1 - beta=80% (nQueryAdvisorR 7.0, [29]). It is expected that the rate of loss to follow-up without recurrence of full-thickness rectal prolapse will add up to 30%. Even though the lack of information on the primary endpoint at 2 years for these patients is partly addressed by the applied time-to-event method, another 30% of patients will be randomized to compensate for this partial loss of information. It can be expected that application of the center-stratified version of the log rank test will further increase the power. Based on these considerations, a total of 130 patients (2 x 65 patients) will be allocated to the trial which should be sufficient to assure a power of 80% for the confirmatory analysis. 

**Table 3:** Literature review for sample size calculation

**Delorme’s procedure**					
**Author*et al.***	**Patients (m/f)**	**Study design**	**Recurrences (%)**	**Improvement incontinence %**	**Improvement obstipation %**
Senapati (1994)	32 (8/24)	Retrospective	12.5	41	16
Oliver (1994)	40 (5/35)	Retrospective	22	68	n.s.
Tobin (1994)	49 (6/43)	Prospective	26	50	n.s.
Lechaux (1995)	85 (8/77)	Retrospective	13.5	69	n.s.
Pescatori (1998)	33	Retrospective	21	n.s.	n.s.
Watts (2000)	101 (10/91)	Retrospective	30	89	44
Watkins (2003)	52 (6/46)	Retrospective	10	58	n.s.
Tsunoda (2003)	31 (7/24)	Retrospective	13	63	38
Marchal (2005)	60 (7/53)	Retrospective	23	42	54
**(Laparoscopic) Resection rectopexy**					
**Author*et al.***	**Patients (m/f)**	**Study design**	**Recurrences (%)**	**Improvement incontinence %**	**Improvement obstipation %**
Huber (1995)	42 (2/40)	Prospective	0	65	41
Stevenson (1998)	30 (1/29)	Prospective	0	70	64
Bruch (1999)	72 (4/68)	Prospective	0	64	76
Kim (1999)	176 (16/160)	Retrospective	5	55	43
Kellokumpu (2000)	34 (3/31)	Prospective	7	n.s.	67
Carpelan (2005)	75 (11/64)	Retrospective	3	n.s.	n.s.
Ashari (2005)	117 (1/116)	Prospective	2.5	62	69
Kariv (2005)	111 (14/97)	Prospective	11	n.s.	n.s.

n.s., Not specified.

#### Analysis

The allocation of all patients to the different analysis populations will be defined prior to the analysis (full analysis set (FAS) according to the intention-to-treat principle (ITT; all randomized patients as randomized), per protocol (PP) analysis set, safety analysis set). The procedure of allocation will be documented in the statistical analysis plan. During the data review, deviations from the protocol will be assessed as ‘minor’ or ‘major’. Major deviations from the protocol will lead to the exclusion of a patient from the PP analysis set.

The confirmatory analysis is performed for the full analysis set. The null-hypothesis ‘no difference in the time to recurrence of full-thickness rectal prolapse between experimental and control intervention during the 2-year follow-up after randomization’ is tested by application of the log rank test stratified by center at the two-sided level α=5%. Drop-out and lost-to-follow-up without recurrence of full-thickness rectal prolapse are considered as censoring events.

For the analysis of the secondary outcomes descriptive methods will be used, including the calculation of appropriate summary methods for the empirical distribution as well as calculation of descriptive two-sided *P* values for comparisons of treatment groups. A special focus of the exploratory analyses will be on the time course of the primary as well as the secondary endpoints. Additionally, sensitivity analyses will be conducted for different study populations (per protocol population, appropriate subgroups). Further exploratory analyses will be performed to identify potential prognostic factors for the intervention effect. The time to recurrence of full-thickness rectal prolapse will additionally be analyzed applying a Cox regression model that includes covariates of potential prognostic value.

The safety analysis is based on all randomized patients who were treated with the interventions under investigation. The analysis includes calculation and comparison of the rates of specified complications (see secondary endpoints) and serious adverse events as well as of severity and relationship to intervention and graphical display of the time course. Furthermore, statistical methods are used to assess the quality of data and the homogeneity of intervention groups.

All analyses will be done using SAS version 9.1 or higher.

### Withdrawals

Participants are allowed to withdraw their written informed consent for the trial at any time and without giving reasons for their decision. If investigators or members of the independent Data Monitoring and Safety Board raise any concerns on an individual patient’s safety, the patient will be withdrawn from the trial.

### Stopping guidelines

The surgical interventions applied in this trial are clinically established standard methods for the treatment of rectal prolapse. Therefore, SAEs that will cause a premature end of the trial are not anticipated.

The DSMB will be informed about the recruitment and relevant safety data in regular time intervals. If any safety concerns arise, the DSMB members will confer on the continuation of the trial and can recommend stopping of the trial. The Ethic Committees then have to be informed.

### Trial organization and administration

#### Funding

DeloRes is funded by the German Research Foundation (Deutsche Forschungsgemeinschaft, DFG).

### Monitoring

Clinical monitoring will be performed by an institution which is independent from other trial staff and experienced due to participation in many other surgical trials. Monitoring procedures will be adapted to the study specific risks for the patients and standard operating procedures (SOP) will be implemented to ensure patients safety and integrity of the clinical data, for example, primary endpoint in adherence to study protocol. Pre-study visits will be performed in centers interested in participating in the study in order to ensure high compliance quality of the participating centers concerning, for example, patient recruitment and documentation. Prior to study start, all participating centers will be personally trained and introduced into all study specific procedures during individual on site initiation visits. Regular on-site monitoring visits are planned at all sites depending on the recruitment rate and quality of the data (approximately one visit per site and year). The investigator must allow the monitor to look at all essential documents and must provide support at all times to the monitor. For all subjects clinical source data verification (SDV) for all clinical items is planned. The extent of further SDV and/or the frequency of monitoring-visits will be adapted for individual centers in case of bad quality of data or if common protocol violations are observed. In return, frequency of monitoring visits can be reduced for reliable and compliant centers with high data quality. In addition to the SOPs all procedures will be predefined in a study-specific monitoring manual. Queries (for example, in case of missing values, implausibility, et cetera) have to be answered by the investigators in a timely manner in order to avoid that errors in data capture or entry are held on to.

### Data and safety monitoring board (DSMB)

To enable an independent risk assessment for the different surgical procedures, potentially related serious adverse events will be noted and periodically assessed by the independent Data and Safety Monitoring Board (DSMB). Detailed working procedures are defined in a different document.

### Steering committee

An independent steering committee will be established. The steering committee will supervise the conduct of the trial and will issue recommendations for early termination, modifications or continuation of the trial, if necessary. The steering committee must be informed in a timely manner of SAE.

#### Ethical and legal aspects

Before the start of the trial, the trial protocol, informed consent document, and any other trial documents were submitted to the independent ethics committee.

The realization of this trial is ethically justifiable since both surgical techniques are routinely used. Study-specific medical interventions exceeding this routine are not planned. Regarding the benefit/risk ratio of this trial for patients, no ethical objections for the realization should exist.

The procedures set out in the trial protocol, pertaining to the conduct, evaluation, and documentation of this trial, are designed to ensure that all persons involved in the trial abide by Good Clinical Practice (GCP) and the ethical principles described in the current revision of the Declaration of Helsinki. The trial will be carried out in keeping with local legal and regulatory requirements.

### Benefit-risk assessment

Benefit-risk assessment will be critically reviewed and has to be approved by the ethics committees in advance before first recruitment.

All interventions applied in this trial represent clinically established, standard methods of treating full-thickness rectal prolapse. Therefore the applicants do not anticipate any serious adverse event that might be triggered due to the patient’s participation in the trial itself.

## Discussion

An evidence-based optimal surgical treatment for all patients with rectal prolapse does not exist [30]. Currently, there is no valid evidence as no conclusive data are yet available from larger randomized or even adequately case-controlled studies. Perineal approaches, such as the Delorme’s procedure, are deemed less invasive compared to abdominal procedures. Further advantages are shorter intervention time and the possibility to perform the operation under spinal or epidural anesthesia. On the other hand, the recurrence rates after such an approach are thought to be significantly higher than after an abdominal approach.

A prospectively randomized, adequately powered trial comparing these two established operation techniques with prolapse recurrence as primary outcome has never been successfully completed. In addition, trial registers of ongoing prospective studies do not indicate that there currently is a recruiting trial investigating this question (last search October 7, 2011).

An updated Cochrane review of Tou *et al*. [18] could only include one RCT comparing a perineal to an abdominal approach in a single center setting with a sample size of 20 and a median follow-up period of 17 months. In this study only one recurrence occurred in the perineal group [31]. An urgent need for high quality randomized clinical trials with adequate statistical power, long-term follow-up, and quality of life assessment is concluded by the authors and reference is made to the PROSPER Trial still ongoing at the time of the Cochrane review update.

The so-called PROSPER Trial in the United Kingdom [32] compared abdominal to perineal surgical approaches for rectal prolapse in a pragmatic two-stage randomization trial design depending on the surgeons degree of uncertainty which of four approaches to choose. The study was able to recruit 293 patients from 33 centers over a 7-year period from February 2001 up to the cessation of the trial in August 2008, but only 26 patients were actually randomized to one of the two approaches (last available newsletter from December 2005, see trial website). All other patients were allocated to one of the two procedures by surgeon’s preference. In view of the very small number of patients actually randomized to a perineal versus an abdominal approach, the conclusions of this study are going to be very limited and the risk of selection bias is high. In addition, the majority of recruiting centers recruited less than 10 patients to the study, further reducing the validity of the results.

Considering the lessons learned from the PROSPER Trial, an urgent need currently exists for an adequately powered, simple parallel-group randomized trial comparing the abdominal to the perineal approach in rectal prolapse. Contrarily to the PROSPER Trial, clinical equipoise regarding the two surgical approaches has been concluded after extensive discussion of the available data as the confidence intervals of the published studies on the two approaches overlap considerably. Even if the study shows that the abdominal procedure results in significantly less recurrences this may be outweighed by a considerably higher morbidity and possibly less improved quality of life. This trial therefore only allows randomization to a perineal or abdominal approach. A loss of eligible patients to an observational non-randomized arm, as was the case for the majority of patients in the PROSPER Trial, will not be possible.

This trial is designed as an expertise-based trial aiming at strong internal validity by high performance and standardization. Generalizability may be reduced by the expertise-based trial design. However, this type of surgery in larger numbers is predominantly performed by specialized surgeons. For these surgeons but also for general surgeons operating only on a few cases per year the results of this trial should have an important impact on clinical practice by determining the most effective treatment for rectal prolapse.

In summary, considering the scarce high-level evidence available up to date we hope that our trial will add the necessary and confirmatory data to analyze which surgical approach is indeed superior.

## Trial status

Recruitment is running since September 2010. All study centers have been initiated. As of March 2012, 186 patients have been screened. As of June 2012, 34 patients have been randomized.

## Competing interests

The authors declare that they have no competing interests.

## Authors’ contributions

SR, FH, PK, SP, AH, and AJ designed and planned the DeloRes Trial. PS and MK supervised the statistical background of the DeloRes Trial. SR, FH, PS, and PK wrote the manuscript. All authors read and approved the final manuscript.

## References

[B1] Ashari LH Lumley JW Stevenson AR Stitz RW Laparoscopically-assisted resection rectopexy for rectal prolapse: ten years’ experience Dis Colon Rectum 2005 48 982 987 10.1007/s10350-004-0886-3 15785889

[B2] Watts AM Thompson MR Evaluation of Delorme’s procedure as a treatment for full-thickness rectal prolapse Br J Surg 2000 87 218 222 10.1046/j.1365-2168.2000.01342.x 10671931

[B3] Bruch HP Herold A Schiedeck T Schwandner O Laparoscopic surgery for rectal prolapse and outlet obstruction Dis Colon Rectum 1999 42 1189 1194 10.1007/BF02238572 10496560

[B4] Kellokumpu IH Vironen J Scheinin T Laparoscopic repair of rectal prolapse: a prospective study evaluating surgical outcome and changes in symptoms and bowel function Surg Endosc 2000 14 634 640 10.1007/s004640000017 10948299

[B5] Lechaux JP Lechaux D Perez M Results of Delorme’s procedure for rectal prolapse. Advantages of a modified technique Dis Colon Rectum 1995 38 301 307 10.1007/BF02055608 7882798

[B6] Marchal F Bresler L Ayav A Zarnegar R Brunaud L Duchamp C Boissel P Long-term results of Delorme’s procedure and Orr-Loygue rectopexy to treat complete rectal prolapse Dis Colon Rectum 2005 48 1785 1790 10.1007/s10350-005-0088-7 15981056

[B7] Kariv Y Delaney CP Casillas S Hammel J Nocero J Bast J Brady K Fazio VW Senagore AJ Long-term outcome after laparoscopic and open surgery for rectal prolapse: a case–control study Surg Endosc 2006 20 35 42 10.1007/s00464-005-3012-2 16374674

[B8] Tsunoda A Yasuda N Yokoyama N Kamiyama G Kusano M Delorme’s procedure for rectal prolapse: clinical and physiological analysis Dis Colon Rectum 2003 46 1260 1265 10.1007/s10350-004-6724-9 12972972

[B9] Oliver GC Vachon D Eisenstat TE Rubin RJ Salvati EP Delorme’s procedure for complete rectal prolapse in severely debilitated patients. An analysis of 41 cases Dis Colon Rectum 1994 37 461 467 10.1007/BF02076192 8181408

[B10] Jorge JM Wexner SD Etiology and management of fecal incontinence Dis Colon Rectum 1993 36 77 97 10.1007/BF02050307 8416784

[B11] Tobin SA Scott IH Delorme operation for rectal prolapse Br J Surg 1994 81 1681 1684 10.1002/bjs.1800811141 7827907

[B12] Watkins BP Landercasper J Belzer GE Rechner P Knudson R Bintz M Lambert P Long-term follow-up of the modified Delorme procedure for rectal prolapse Arch Surg 2003 138 498 502 10.1001/archsurg.138.5.498 12742952

[B13] Pescatori M Interisano A Stolfi VM Zoffoli M Delorme’s operation and sphincteroplasty for rectal prolapse and fecal incontinence Int J Colorectal Dis 1998 13 223 227 10.1007/s003840050165 9870165

[B14] Stevenson AR Stitz RW Lumley JW Laparoscopic-assisted resection-rectopexy for rectal prolapse: early and medium follow-up Dis Colon Rectum 1998 41 46 54 10.1007/BF02236895 9510310

[B15] Kuhry E Schwenk W Gaupset R Romild U Bonjer J Long-term outcome of laparoscopic surgery for colorectal cancer: a cochrane systematic review of randomised controlled trials Cancer Treat Rev 2008 34 498 504 10.1016/j.ctrv.2008.03.011 18468803

[B16] Sajid M Siddiqui M Baig M Open vs laparoscopic repair of full-thickness rectal prolapse: a re-meta-analysis Colorectal Dis 2009 13 515525 10.1111/j.1463-1318.2009.01886.x 20557324

[B17] Sammour T Kahokehr A Srinivasa S Bissett IP Hill AG Laparoscopic colorectal surgery is associated with a higher intraoperative complication rate than open surgery Ann Surg 2011 253 35 43 10.1097/SLA.0b013e318204a8b4 21294286

[B18] Tou S Brown SR Malik AI Nelson RL Surgery for complete rectal prolapse in adults Cochrane Database Syst Rev 2008 8 CD001758 18843623 10.1002/14651858.CD001758.pub2

[B19] Dindo D Demartines N Clavien PA Classification of Surgical complications: A new Proposal with evaluation in a cohort of 6336 Patients and results of a survey Ann Surg 2004 240 205 213 10.1097/01.sla.0000133083.54934.ae 15273542 PMC1360123

[B20] Rahbari NN Weitz J Hohenberger W Heald RJ Moran B Ulrich A Holm T Wong WD Tiret E Moriya Y Laurberg S den Dulk M van de Velde C Büchler MW Definition and grading of anastomotic leakage following anterior resection of the rectum: a proposal by the International Study Group of Rectal Cancer Surgery 2010 147 339 351 10.1016/j.surg.2009.10.012 20004450

[B21] Horan TC Gaynes RP Martone WJ Jarvis WR Emori TG CDC definitions of nosocomial surgical site infections, 1992: a modification of CDC definitions of surgical wound infections Infect Control Hosp Epidemiol 1992 13 606 608 10.1086/646436 1334988

[B22] Ware J Kosinski M Keller SD A 12-Item Short-Form Health Survey: construction of scales and preliminary tests of reliability and validity Med Care 1996 34 220 233 10.1097/00005650-199603000-00003 8628042

[B23] Braun M Herold A Krammer H Schlieger F Evaluierung eines Scores zur Unterscheidung verschiedener Obstipationsformen Coloproctology 2006 28

[B24] Rockwood TH Church JM Fleshman JW Kane RL Mavrantonis C Thorson AG Wexner SD Bliss D Lowry AC Patient and surgeon ranking of the severity of symptoms associated with fecal incontinence: the fecal incontinence severity index Dis Colon Rectum 1999 42 1525 1532 10.1007/BF02236199 10613469

[B25] Senapati A Nicholls RJ Thomson JP Phillips RK Results of Delorme’s procedure for rectal prolapse Dis Colon Rectum 1994 37 456 460 10.1007/BF02076191 8181407

[B26] Kim DS Tsang CB Wong WD Lowry AC Goldberg SM Madoff RD Complete rectal prolapse: evolution of management and results Dis Colon Rectum 1999 42 460 466 10.1007/BF02234167 10215045

[B27] Carpelan-Holmström M Kruuna O Scheinin T Laparoscopic rectal prolapse surgery combined with short hospital stay is safe in elderly and debilitated patients Surg Endosc 2006 20 1353 1359 10.1007/s00464-005-0217-3 16703440

[B28] Huber FT Stein H Siewert JR Functional results after treatment of rectal prolapse with rectopexy and sigmoid resection World J Surg 1995 19 138 143 10.1007/BF00316999 7740801

[B29] nQuery Advisor® Release 7.0. Statistical Solutions Saugus http://www.statistical-solutions-software.com/products-page/nquery-advisor-sample-size-software

[B30] Schiedeck TH Schwandner O Scheele J Farke S Bruch HP Rectal prolapse: which surgical option is appropriate? Langenbecks Arch Surg 2005 390 8 14 10.1007/s00423-004-0459-x 15004753

[B31] Deen KI Grant E Billingham C Keighley MRB Abdominal resection rectopexy with pelvic floor repair versus perianal rectosigmoidectomy and pelvic floor repair for full-thickness rectal prolapse Br J Surg 1994 81 302 304 10.1002/bjs.1800810253 8156369

[B32] PROSPER - PROlapse Surgery PErineal or Rectopexyhttp://www.prosper.bham.ac.uk

